# Electroesophagogram in gastroesophageal reflux disease with a new theory on the pathogenesis of its electric changes

**DOI:** 10.1186/1471-2482-4-13

**Published:** 2004-10-05

**Authors:** Ahmed Shafik, Olfat El-Sibai, Ismail Shafik, Ali Shafik

**Affiliations:** 1 Department of Surgery and Experimental Research, Faculty of Medicine, Cairo University, Cairo, Eygpt; 2 Department of Surgery, Faculty of Medicine, Menoufia University, Shebin El-Kom, Eygpt

**Keywords:** Slow waves, action potentials, acid reflux, GERD, gastroesophageal reflux disease

## Abstract

**Background:**

In view of the disturbed esophageal peristaltic activity and abnormal esophageal motility in gastroesophageal reflux disease, (GERD), we investigated the hypothesis that these changes result from a disordered myoelectric activity of the esophagus.

**Methods:**

The electric activity of the esophagus (electroesophagogram, EEG) was studied in 27 patients with GERD (16 men, 11 women, mean age 42.6 ± 5.2 years) and 10 healthy volunteers as controls (6 men, 4 women, mean age 41.4 ± 4.9 years). According to the Feussner scoring system, 7 patients had a mild (score 1), 10 a moderate (score 2) and 10 a severe (score 3) stage of the disease. One electrode was applied to the upper third and a second to the lower third of the esophagus, and the electric activity was recorded. The test was repeated after the upper electrode had been moved to the mid-esophagus.

**Results:**

The EEG of the healthy volunteers showed slow waves and exhibited the same frequency, amplitude and conduction velocity from the 2 electrodes of the individual subject, regardless of their location in the upper, middle or lower esophagus. Action potentials occurred randomly. In GERD patients, score 1 exhibited electric waves' variables similar to those of the healthy volunteers. In score 2, the waves recorded irregular rhythm and lower variables than the controls. Score 3 showed a "silent" EEG without waves.

**Conclusion:**

The electric activity in GERD exhibited 3 different patterns depending on the stages of GERD. Score 1 exhibited a normal EEG which apparently denotes normal esophageal motility. Score 2 recorded irregular electric waves variables which are presumably indicative of decreased esophageal motility and reflux clearance. In score 3, a "silent" EEG was recorded with probably no acid clearance. It is postulated that the interstitial cells of Cajal which are the electric activity generators, are involved in the inflammatory process of GERD. Destruction of these cells appears to occur in grades that are in accordance with GERD scores. The EEG seems to have the potential to act as an investigative tool in the diagnosis of GERD stages.

## Background

Reflux esophagitis is a multifactorial disease that entails the reflux of gastric contents into the esophageal lumen [1]. Esophagitis develops when noxious substances in the refluxate have sufficient time to get in contact with the esophageal mucosa and then prevail over structural and functional defenses [2] The mechanisms involved in the defense of the esophagus against the gastric acid pepsin comprise the antireflux barriers, luminal clearance and epithelial resistance [3-6]. The antireflux barriers and the luminal clearance mechanism by peristalsis represent motor components of the reflux disease. The non-motility elements include salivary and esophageal submucosal glands.

Abnormal esophageal motility results in an increased exposure of the esophageal mucosa to gastric contents. Progressing severity of the reflux disease is associated with failing primary peristalsis [7-9]. The failure rate in patients with mild and severe GERD is 25% and 36%, respectively [7]. The amplitude of peristaltic contractions in the esophagus is significantly lower in the esophagitis patients than in the controls [6-9], and is inversely related to esophagitis severity [7,10]. The duration of contractions was variable but the propagation velocity was unequivocally slower in esophagitis patients than in controls [7,8,11].

Previous studies have shown that the esophagus possesses electric activity presenting as slow waves (SWs) followed or superimposed by fast activity spikes or action potentials (APs) [12,13]. Action potentials were associated with a rise in the intraesophageal pressure. Balloon distension of the esophagus effected an increase in the electric activity proximally to the balloon and a decrease distally [12]. The caudad direction of the SWs and APs was evidenced when after esophageal myotomy the potentials appeared proximally but not distally to the cut in the experimental animal [12]. This suggested also the presence of a pacemaker in the cervical esophagus which might initiate the electric waves[12].In achalasia of the esophagus, three electroesophagographic patterns were identified: bradyesophagia, esophagoarrhythmia and "silent" electroesophagogram [13]. The three patterns seem to represent different stages in one pathologic process.

In view of the disturbed esophageal peristaltic activity and abnormal esophageal motility in GERD, we hypothesized that these findings result from a disordered myoelectric activity of the esophagus. This hypothesis was investigated in the current study.

## Methods

### Subjects

Twenty seven patients with GERD (16 men, 11 women, mean age 42.6 ± 5.2 SD years, range 36–48) were enrolled in the study. The diagnosis was confirmed by 24-hour pH monitoring, endoscopy and esophageal motility test. According to Feussner's scoring system [14], seven patients had a mild (score1), 10 a moderate (score 2) and 10 a severe (score 3) stage of the disease. Score 1 had mild heartburn with mild chest pain but no regurgitation, dysphagia, hiatus hernia or mucosal changes. Exposure time to pH was <4.4–8%. Score 2 had moderate heartburn, moderate chest pain, regurgitation after large meals, small hiatus hernia, and isolated erosive mucosal lesions. Score 3 had severe heartburn, severe chest pain, regurgitation, occasional dysphagia, hiatus hernia, and esophageal ulcers.

The study also included 10 healthy volunteers (6 men, 4 women, mean age 41.4 ± 4.9 SD years, range 35–50) who had no reflux esophagitis. They had no gastrointestinal complaints in the past or at the time of enrollment in the study.

Physical examination of both the patients and healthy volunteers had normal findings. The results of laboratory work comprising blood count, renal and hepatic function tests as well as electrocardiogram were unremarkable. The studied subjects gave an informed consent after having been fully informed about the nature of the study, the tests to be done and their role in the study. The study was approved by the Review Board and Ethics Committee of the Cairo University Faculty of Medicine.

### Methods

The electric activity of the esophagus was recorded in the patients with GERD and the healthy volunteers. We used a monopolar silver-silver chloride electrodes of 0.8 mm diameter (Smith-Kline Beecham, Los Angeles, CA, USA) introduced through a 6F catheter (Rubber Industries Ltd., London, UK) with the electrode protruding by 1 cm from the catheter tip. The catheter was attached to the esophageal mucosa by negative pressure suction of 50 to 100 mmHg which was maintained during the test.

Two electrodes were introduced into the esophagus by means of an endoscope and fixed to the esophageal mucosa by suction; one electrode was applied to the upper third, and the second to the lower third of the esophagus, and the electric activity was recorded. The upper electrode was then transferred to the middle third of the esophagus and recordings of the electric activity from the middle and lower third electrodes were performed. Signals from the electrodes were fed into an AC amplifier with a frequency response within ± 3 dB from 0.016 Hz to 1 kHz; they were displayed on a recorder at a sensitivity of 1 mV/cm. A metal disc applied to the abdominal skin served as the indifferent electrode. A strain gauge respiration transducer was attached to the thoracic wall for respiratory artefacts. We allowed the esophagus a 30 minute period to adapt to the electrodes applied to its wall, before we started a 120-min recording session for each subject.

The results were analyzed statistically using the Student's t test. Differences assumed significance at p < 0.05 and values were given as the mean ± standard deviation (SD).

## Results

No adverse side effects were encountered during or after the performance of the tests and all the subjects were evaluated. There was no difficulty in applying the electrodes to the esophageal mucosa. We found that a suction pressure of 50–60 mmHg was sufficient to keep the electrode fixed to the esophageal mucosa during the testing period in most of the subjects; in few cases we had to increase the suction pressure to 100 mm to keep the electrodes in position. Applying the aforementioned pressures, we encountered neither migration nor detachment of the electrodes during the entire test. We met no mucosal bleeding, tears or ulcers during application or after removal of the electrodes either.

### Electroesophagogram in healthy subjects

**Figure 1:**
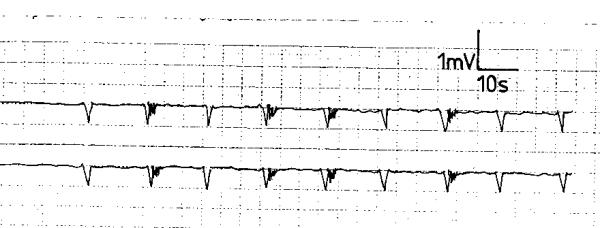
Electroesophagogram of a healthy volunteer showing slow waves with regular rhythm and random action potentials.

Monophasic negatively deflected SWs were recorded from the 2 electrodes of each subject of all the studied individuals (fig. 1). They had an unvariable shape in all recordings from the same site. The frequency, amplitude and conduction velocity were constant in the individual subject. The SWs in each individual exhibited the same frequency, amplitude and regular rhythm from both electrodes (fig. 1), regardless of their location in the upper, middle or lower third of the esophagus. The mean and range of frequency, amplitude and conduction velocity of the 10 healthy volunteers are displayed in table 1. These values were reproducible from the electrodes in the upper, middle or lower third of the esophagus. Bursts of APs representing fast activity spikes were recorded (fig. 1). They followed or were superimposed over the SWs; they occurred randomly and their frequency was inconsistent in each subject.

**Table 1:** The frequency, amplitude and conduction velocity of the slow waves of the healthy volunteers and patients with gastroesophageal reflux disease (GERD)^+^

	**Slow waves**					
	
	**Frequency c/m**		**Amplitude (mV)**		**Velocity (cm/s)**	
	
	**Mean**	**Range**	**Mean**	**Range**	**Mean**	**Range**
**Volunteers**	5.2 ± 1.3	4 – 7	0.52 ± 0.1	0.4 – 0.6	4.8 ± 0.7	3.5 – 6.1
**Score 1 GERD**	4.8 ± 1.2	4 – 6^•^	0.49 ± 0.1^•^	0.35 – 0.6	4.6 ± 0.7^•^	4.1 – 5.9
**Score 2 GERD**	Irregular					
**Score 3 GERD**	Absent waves					

^+ ^values were given as the mean ± SD

^• ^p > 0.05

p values of the patients were compared to those of the healthy volunteers

### Electroesophagogram in GERD patients

**Figure 2:**
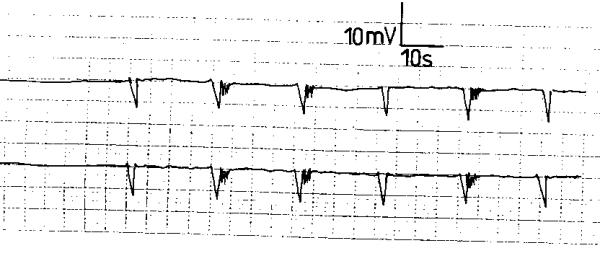
Electroesophagogram of a patient with score 1 gastroesophageal reflux disease showing slow waves with regular rhythm and random action potentials.

In score 1 GERD, the electric waves' variables were similar to those of the healthy volunteers in all the subjects (p > 0.05, fig 2). The SWs were monophasic and negatively deflected and had a regular rhythm (fig 2). The frequency, amplitude and conduction velocity are shown in table 1. Action potentials were randomly recorded following or superimposed over the SWs. This electromyographic pattern was similar from the 2 recording electrodes of the individual subject and was reproducible during the recording period.

**Figure 3:**
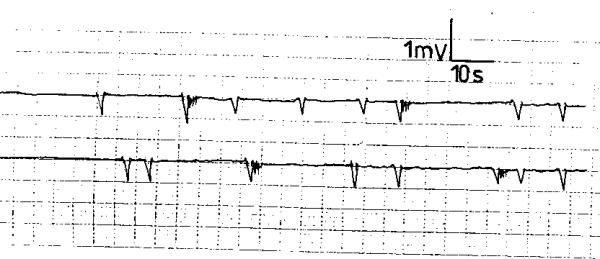
Electroesophagogram of a patient with score 2 gastroesophageal reflux disease exhibiting slow waves with irregular rhythm and varying frequency, amplitude and conduction velocity from the same electrode and between the 2 electrodes of the same subject.

**Figure 4:**
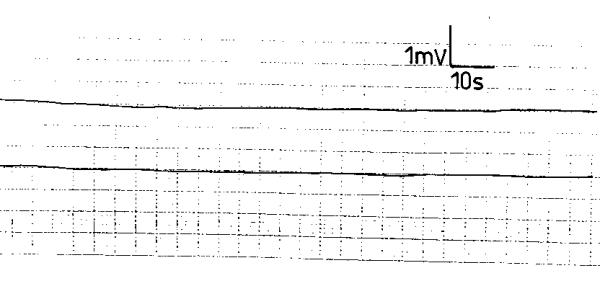
Electroesophagogram of a patient with score 3 gastroesophageal reflux disease recording no electric activity: a "silent" electroesophagogram.

**Figure 5:**
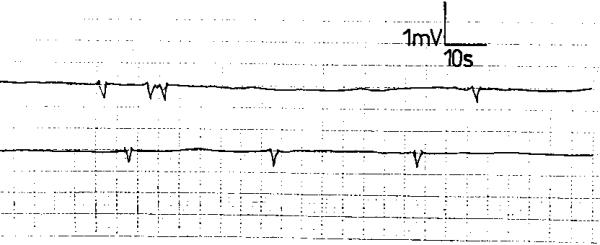
Electroesophagogram of a patient with score 3 gastroesophageal reflux disease recording occasional slow waves with no action potentials.

Score 2 GERD patients exhibited a different electromyographic pattern. The SWs had an irregular rhythm with varying but lower frequency, amplitude and conduction velocity compared to the normal controls (fig. 3). The APs occurred randomly and were less frequent than in the normal recordings (fig. 3). The SWs and APs differed from one electrode to the other of the same subject and were variable during the recording period. In 8 score 3 GERD patients, the electrodes did not register electric waves; neither SWs nor APs were recorded. A "silent" electromyographic pattern was registered (fig. 4); this picture was reproducible during the recording period. In the remaining 2 patients; occasional SWs were recorded that were inconsistent and different from the 2 electrodes of the same patient (fig. 5); no APs were recorded at any time during the recording period (fig. 5).

## Discussion

The current study has demonstrated that the esophagus possesses an electric activity in the form of regular SWs and APs. The waves in the healthy volunteers were reproducible with identical frequency, amplitude and conduction velocity in the same subject. Previous studies have shown that the APs were coupled with increased esophageal pressure, while the SWs were not [12,13], these findings presumably denote that the APs have a contractile activity [12,13].

Effective esophageal motility is a critical determinant for esophageal clearance of refluxed gastric contents [15]. A single normal peristaltic wave completely clears the entire barium bolus from the esophagus. If a peristaltic wave fails due to motile dysfunction, there is little or no volume clearance [16]. Increased exposure of the esophageal mucosa to gastric contents may result from abnormal esophageal motility. Thus, while a defective gastroesophageal barrier accounts for an increased number of gastroesophageal reflux episodes, abnormal esophageal peristalsis results in impaired esophageal acid clearance [17,18].

The relationship between esophageal peristalsis and gastroesophageal reflux has been studied using stationary and ambulatory prolonged esophageal manometry. Various motor-disorders have been detected in GERD including a significantly high rate of incomplete primary peristalsis, changes in esophageal motility and an increased number of non-transmitted contractions [6,19]. Contractions had a shorter duration and a slower propagation velocity [20].

Patients with abnormal GER but mild esophagitis, or none, had normal amplitude of contractions with increased prevalence of simultaneous contractions [16,21]. Meanwhile patients with severe esophagitis had reduced amplitude of contractions, slow propagation velocities and an increased rate of failed primary peristalsis [21]. The current study may shed some light on the mechanism of these motor changes in GERD.

The electric activity in GERD exhibited different patterns depending on the stage of the GERD. In score 1 GERD, an electroesophagram similar to that of healthy volunteers was recorded. This apparently denotes that the motile activity of the esophagus in score 1 is normal and that the esophageal peristaltic activity can probably clear the esophagus of the refluxed gastric contents. The irregular and diminished esophageal electric wave variables displayed in score 2 GERD are presumably indicative of diminished motile activity and peristalsis of the esophagus with a resulting inhibited reflux clearance rate. The failure of adequate esophageal clearance is probably responsible for the clinical manifestations and investigative results encountered in score 2 GERD. With progress of the condition to score 3, there is probably no motor or peristaltic esophageal activity as evidenced by the absence of the esophageal electric waves. In such case, we presume that there is no esophageal clearance.

We do not know the cause of the diminished esophageal electric activity in GERD. Is it due to the refluxed acid material or to the resulting esophageal inflammation? It may be argued that the refluxed acid content into the esophagus inhibits its motile and peristaltic activity. However, the current and earlier studies have demonstrated normal peristalsis in score 1 GERD in which acid reflux was manifest [16]. Furthermore, the current study showed normal EMG activity in this condition. Probably these findings negate the role of acid reflux as inhibitor of the peristaltic and electric activity in GERD.

What then could be the cause of the deranged electric activity and peristaltic movement in the more advanced stages of GERD?

### A new theory of the pathogenesis of diminished electric activity in GERD

The electric waves seem to be generated from the interstitial cells of Cajal that are located at the level of the myenteric plexus and in the circular muscle layer of the esophageal wall [22-24]. They are considered as the generators of the spontaneous pacemaker activity in the smooth muscle layers of the gut [22-24] and are also involved in neurotransmission [25-27]. They mediate or transduce inputs from enteric motor nerves to the smooth muscle syncytium.

In the advanced stages of GERD it may be assumed that the inflammatory changes in the esophageal wall have involved the interstitial cells of Cajal. Destruction of these cells appears to occur in grades that are in accordance with the GERD socres. It seems that in score 1 GERD, the mucosal inflammatory changes of the esophagus, if present, have not involved the Cajal cells yet. With the more advanced stages of the disease as in scores 2 and 3, the Cajal cells are presumingly being gradually destroyed by the advancing esophageal inflammatory process. The diminished SW variables encountered in score 2 GERD seem to be due to partial involvement of Cajal cells in the inflammatory process; the cells are not yet completely destroyed and are still mediating electric and peristaltic activity. However the Cajal cells in score 3 GERD are believed to be extremely injured so that they cannot generate electric activity.

### Diagnostic role of electroesophagogram in GERD

There are various methods for the diagnosis of GERD. They include pressure measurements using water-perfused manometry catheters, external transducers or intraluminal transducers, pH-metry and others [28,29]. However they might have disadvantages [30]. In view of the results of our above study, the introduction of the electroesophagogram as an investigative tool may be a valuable addition to the armamentarium of esophageal investigations of GERD.

## Conclusion

The electric activity in GERD expressed 3 different patterns depending on the stage of GERD. In score 1 GERD, a normal electroesophagogram was recorded which would denote normal esophageal motile activity and acid clearance. Score 2 GERD exhibited irregular and diminished esophageal electric waves' variables which are presumably indicative of decreased esophageal motility and reflux clearance. In score 3, a "silent" electroesophagogram was recorded with probably no acid clearance. A new theory of the pathogenesis of diminished electric activity in GERD is put forward. It is postulated that the interstitial cells of Cajal which are considered as the generators of the pacemaker activity and electric waves, are involved in the inflammatory process of GERD. Destruction of these cells appears to occur in grades that are in accordance with GERD scores. The electroesophagogram may serve as an investigative tool in diagnosing the various GERD stages, especially if they can be recorded percutaneously.

## List of abbreviations

gastroesophageal reflux disease = GERD

(electroesophagogram = EEG

slow waves = SWs

action potentials = APs

standard deviation = SD

## Authors contributions

AS: Study design/ planning

OES: Data collection/entry, Data analysis/statistics, Literature analysis/search

IS: Data collection/entry, Data analysis/statistics, Literature analysis/search

AS: Data collection/entry, Preparation manuscript

## Pre-publication history

The pre-publication history for this paper can be accessed here:


